# Management of cervical artery dissection: new evidence and future directions

**DOI:** 10.1007/s00415-025-13166-1

**Published:** 2025-05-27

**Authors:** Josefin E. Kaufmann, Lukas Mayer-Suess, David Seiffge, Michael Knoflach, Stefan T. Engelter, Christopher Traenka

**Affiliations:** 1 Department of Neurology and Stroke Center, University Hospital Basel and University of Basel, Basel, Switzerland; 2 Department of Rehabilitation and Neurology, Department of Geriatric Medicine FELIX PLATTER, University, University of Basel, Basel, Switzerland; 3 Department of Neurology, Medical University of Innsbruck, Innsbruck, Austria; 4 Department of Neurology, Inselspital Bern, Bern, Switzerland; 5 Research Centre On Clinical Stroke Research, VASCage, Innsbruck, Austria

**Keywords:** Cervical artery dissection, Stroke, Mental health, Antithrombotic treatment, Vascular risk factors, Hyperacute treatment

## Abstract

Cervical artery dissection (CeAD) is a leading cause of ischemic stroke in young adults. Although its pathogenesis remains incompletely understood, advancements in CeAD patient care have been made in recent years. This review provides an updated overview of the latest evidence on hyperacute and (sub-)acute management of CeAD, highlighting aspects that have received limited attention, including vascular risk factors and mental health. Furthermore, we aim to outline future research directions to enhance patient outcomes and deepen our understanding of the disease.

## Introduction

Cervical Artery Dissection (CeAD) is a leading cause of ischemic stroke in young adults [1], occurring at a mean age of 45 years [2]. However, its pathogenesis remains incompletely understood [3, 4]. CeAD is most commonly hypothesized to result from an interaction of individual (e.g., hereditary predisposition [2, 4, 5]), and environmental factors (e.g., infection [6, 7], trauma [8, 9]), connective tissue alterations [10–12], and comorbidities (such as migraine [13, 14] and hypertension [15]). All of these factors are thought to contribute to arterial wall weakness or act as triggers, thereby increasing susceptibility to CeAD. [3]

Fortunately, despite the limited understanding of its underlying mechanisms, knowledge of CeAD patient care has seen an incremental increase over the past two decades. This review highlights new evidence in the hyperacute and (sub-)acute treatment of CeAD. Additionally, it identifies gaps in current CeAD management that have hitherto been overlooked, such as the value of vascular risk factor treatment and the inclusion of mental health in long-term post-CeAD care. These areas represent key opportunities for future research and will be essential to improve patient-relevant CeAD outcomes.

## New approaches in hyperacute and (sub-)acute management of CeAD

### Intravenous thrombolysis

The use of intravenous thrombolysis (IVT) in CeAD has long been based on CeAD subgroup analyses of large IVT registries and observational studies showing no significant functional benefit [16–20]. Therefore, according to the 2021 ESO guidelines, IVT for ischemic CeAD is considered a low-evidence intervention [20]. This lack of evidence prompted two studies, published in 2024 and 2025, to investigate the efficacy and safety of IVT in patients with CeAD involving both anterior and posterior circulation. Shu et al. analyzed the observational STOP-CAD dataset (*n* = 1653, 90-day modified Rankin Scale [mRS] available for *n* = 1304) and found that IVT-treated patients (*n* = 414) compared to those not receiving IVT (*n* = 890) had higher odds of achieving functional independence (measured by a modified Rankin Scale [mRS] 0–2; adjusted OR 1.67, 95% CI 1.22–2.28, *p* = 0.001), but not with symptomatic intracranial hemorrhage (ICH) (adjusted OR = 1.52, 95% CI 0.79–2.92, *p* = 0.215). The rate of symptomatic ICH was 4.1% in IVT-treated patients, compared to 1.6% in those who did not receive IVT [21]. Furthermore, the benefit of IVT was not observed in subgroups including females, patients with mild strokes, those with partially occlusive thrombi, or with intracranial extension of the dissection – findings that should be interpreted cautiously due to possibly limited power in the subgroup analyses [21]. These main findings were supported by a U.S. claims database study that included 11,285 patients with ischemic CeAD, of whom 1,360 (12.1%) received IVT. Adjusted analyses showed IVT was associated with higher odds of home discharge (adjusted OR 1.40, 95% CI 1.01–1.92, *p* = 0.042), but not with ICH (adjusted OR 0.69, 95% CI 0.32–1.48, *p* = 0.341), despite IVT-treated patients having higher baseline stroke severity (median National institutes of Health Stroke Scale scores (NIHSS) [interquartile range], 8 [4–17], versus 3 [1–11], *p* = < 0.001). The ICH rate in the IVT group was 4% [22]. Of note, these ICH rates in IVT-treated CeAD patients are consistent with those reported in previous thrombolysis studies for non-CeAD acute ischemic stroke. [23, 24]

These two CeAD studies [21, 22] are the first to demonstrate a potential benefit of IVT in patients with acute ischemic stroke due to CeAD. However, as they are based on observational data, cautious interpretation is warranted. Given the established efficacy of IVT in acute ischemic stroke overall, IVT should not be withheld from CeAD patients treated on-label. [25]

### Endovascular treatment

In line with IVT, endovascular treatment (EVT) for CeAD patients is considered a very-low-evidence intervention in the current ESO guidelines [19, 20, 26–29]. Although EVT has been associated with higher recanalization rates compared to IVT, this has not consistently translated into improved functional outcomes [29, 30]. This discrepancy may be partly explained by selection bias, as patients undergoing EVT often present with higher baseline NIHSS scores [26, 29, 31]. In this context, the study by Li et al. is noteworthy: using propensity score matching, the authors compared 48 patients with CeAD who received EVT to 48 patients who did not, thereby balancing baseline stroke severity. This study demonstrated a significant benefit of EVT, with 66.7% of patients achieving a good functional outcome at 90 days compared to 39.6% in the non-EVT group. Since the publication of the current guidelines, a recent study provides new insights.

The CONCORDIA study, a collaborative stroke registry analysis looking at data from Austria, Germany, and Switzerland (*n* = 1023, 516 receiving EVT), analyzed outcomes in CeAD patients with angiographically proven anterior circulation large vessel occlusion who underwent EVT [32, 33]. The study found a significant benefit of endovascular treatment in achieving a favorable 3-month functional outcome (measured by mRS 0–2; adjusted relative risk [RR] 1.29, 95% CI 1.19–1.41). As a limitation, the mRS was available for only 409 (73.9%) of EVT-treated patients and 398 (78.5%) of those receiving best medical management. However, EVT was also associated with a higher risk of intracranial hemorrhage (adjusted RR 3.50, 95% CI 1.56–7.82) compared to best medical management [32, 33]. Notably, in patients with an NIHSS < 6, EVT was associated with worse functional outcomes and higher rates of symptomatic intracranial hemorrhages compared to best medical treatment [32, 33]. These findings align with recent studies on endovascular treatment in low NIHSS strokes independent of underlying etiology [34]. Therefore, based on this sole large observational registry study, EVT appears to be a reasonably beneficial treatment option in patients with CeAD, large vessel occlusion, and NIHSS of ≥ 6. [32, 33]

Evidence on the use of EVT for posterior circulation involvement in CeAD-related large-vessel occlusion strokes remains limited. However, two studies comparing EVT outcomes between anterior and posterior circulation large vessel occlusion strokes in the context of CeAD reported no significant differences in functional outcomes, reperfusion rates, or adverse events [35, 36]. Although these findings are based on very small sample sizes (posterior circulation was affected in *n* = 10 [35] and *n* = 17 [36] patients, respectively), they support the notion that EVT may be similarly safe and effective in posterior circulation CeAD-related large vessel occlusion. In light of this, neurologists and neurointerventionalists can consider EVT on an individual patient basis, despite the limited available evidence.

### Stenting of the internal carotid artery

The optimal management of extra-intracranial tandem occlusions in patients with CeAD remains debated. In the hyperacute setting, there are currently no evidence-based guidelines or consensus on the indications for carotid artery stenting in the context of CeAD. STOP-CAD emerged with an analysis of 328 patients with occlusive internal carotid artery dissection and anterior circulation ischemic stroke, of which 150 underwent emergent stenting of the extracranial (i.e., CeAD-related) pathology. The median age was 51 (IQR 44–58) years, and 96 patients (29.3%) were female. At 90 days, no significant difference in functional outcomes (mRS available in 84% of the patients; 62.0% versus 59.7%; adjusted OR 1.23, 95% CI 0.82–1.86, *p* = 0.315) or symptomatic ICH rates was observed (7.3% versus 7.9%; adjusted hazard ratio [HR] 1.69, 95% CI 0.01–240.6, *p* = 0.836) [37]. However, in the acute phase (i.e., 24 h after endovascular assessment), patients who underwent stenting had a significantly higher risk of any intracranial hemorrhage (adjusted OR 2.02, 95% CI 1.11–3.67, *p* = < 0.001), which was not significant for symptomatic ICH alone (adjusted OR 0.95, 95% CI 0.41–2.2, *p* = 0.913) [37]. These results suggest that while both treatment strategies are viable, emergent stenting may have a less favorable safety profile due to an increased risk of early hemorrhage, which is consistent with the results of other observational studies [28, 38–41]. Still, in cases of hemodynamic instability, where cerebral perfusion imaging indicates a potential benefit from restoring blood flow, emergent stenting can be considered [42, 43]. Ultimately, the decision to use emergent stenting remains an individual decision made by the treating physician, taking into account the specific clinical circumstances and patient factors. Given the rarity of CeAD, randomized controlled trials on acute stenting are unlikely to be feasible. Nevertheless, future observational studies or registry-based analyses could help identify subgroups who might benefit, particularly patients with hemodynamic instability, as evidenced by delayed time-to-peak on perfusion imaging or gradual clinical worsening through hypoperfusion-related recurrent cerebral ischemia. Comparing outcomes in such patients with and without stenting could provide valuable insights into the role of revascularization in this setting. The ongoing TITAN trial (Thrombectomy in Tandem Occlusion) will shed more light on further safety and efficacy of emergency stenting in tandem occlusion. [24]

### Antithrombotic treatment—antiplatelets or anticoagulation?

Uncertainty persists regarding the optimal antithrombotic therapy to best prevent (subsequent) cerebral ischemic events in patients with CeAD. Despite considerable efforts, including two pivotal randomized controlled trials (RCTs)—the Cervical Artery Dissection in Stroke Study (CADISS) [44–46] and the Biomarkers and Antithrombotic Treatment in Cervical Artery Dissection (TREAT-CAD) [47, 48]—no clear consensus has yet emerged. Both trials compared anticoagulation through vitamin K antagonists (VKA) with primarily single antiplatelet therapy (some patients in CADISS were treated with dual antiplatelets) [44, 48]. Neither the individual trials [45–47] nor the study-level meta-analyses published in the 2021 ESO guidelines [20] were able to definitively reveal a superior treatment, leaving this clinically important question unanswered. However, recent studies have put forth valuable insights for potential future endeavors.

An individual participant data meta-analysis combining data from the CADISS and TREAT-CAD trials showed a non-significant reduction in the composite primary endpoint—ischemic stroke and major bleeding—among participants randomized to anticoagulation (3 of 218 [1.4%] vs 10 of 226 [4.4%]; OR 0.33, 95% CI 0.08–1.05, *p*-value = 0.06) [49]. Additionally, when focusing solely on ischemic stroke as an outcome, anticoagulation showed a significant benefit (1 of 218 [0.5%] vs 10 of 226 [4.0%]; OR 0.14, 95% CI 0.02–0.61, *p*-value = 0.01), suggesting a potential advantage of anticoagulation in the setting of stroke prevention [49]. However, it should be noted that all serious bleeding events occurred in the anticoagulated patients compared to those treated with antiplatelets (*n* = 2 versus *n* = 0). [49]

The STOP-CAD observational study recently added safety aspects to the discussion by comparing anticoagulation (including both vitamin K antagonists and direct oral anticoagulants [DOACs]) with antiplatelet therapy (both single and dual regimens) in 3,636 patients with CeAD [50]. While there was no significant difference between the two treatment groups in preventing ischemic stroke at six months post-CeAD-diagnosis (adjusted HR 0.80, 95% CI, 0.28–2.24, *p*-value = 0.67) [50], anticoagulation was significantly associated with an increased risk of major hemorrhagic events (adjusted HR 5.56, 95% CI 1.53–20.13, *p*-value = 0.009). [50]

A comprehensive systematic review and meta-analysis integrating data from all three landmark studies (CADISS, TREAT-CAD, and STOP-CAD) was recently published [51]. Herein, taking together both the promising results in stroke prevention of the CADISS and TREAT-CAD individual participant data meta-analysis and the safety concerns of STOP-CAD [51]. In this analysis, anticoagulation significantly outperformed antiplatelet therapy in preventing ischemic strokes (RR 0.63, 95% CI 0.43–0.94, *p*-value = 0.02, *I*-squared = 0%) with a number needed to treat of 50 [51]. Conversely, anticoagulation was associated with a higher risk of major hemorrhage (RR 2.25, 95% CI 1.07–4.72, *p*-value = 0.03, *I*-squared = 0%), which corresponds to a number needed to harm of 135 [51]. In conclusion, anticoagulation may confer a net clinical benefit, despite an increased bleeding risk [51]. While caution is warranted, as the significant results of the meta-analysis were largely driven by the sample size of the observational STOP-CAD study [51], it is reassuring that these pooled data analyses demonstrated a net benefit for anticoagulation. Still, the current evidence for anticoagulation is unlikely to translate into routine CeAD care, as considerable limitations call for caution in generalizing and interpreting the results. This highlights the need for additional RCTs to provide the highest level of evidence for either treatment strategy. Future trials should also incorporate modern antithrombotic agents, such as dual antiplatelet therapy and DOACs, to align with current clinical practice [49] and the expert consensus statement from the ESO guidelines. [20]

The current evidence on the role of DOACs in patients with CeAD remains limited. A systematic review and meta-analysis from 2022 included 699 patients treated with VKAs and 53 with DOACs, finding comparable efficacy in preventing TIA/stroke (VKA: 12.3%, 95% CI 0–28.6%; DOAC: 5.7%, 95% CI 0–12.2%) and safety in preventing intracranial bleeding (VKA: 1.2%, 95% CI 0.3–2%; DOAC: 8.6%, 95% CI 0–34.4%) [52]. However, the small sample size of DOAC-treated patients limits the generalizability [52]. Further analyses, such as the planned substudy of the large observational STOP-CAD study, are therefore eagerly awaited.

### Antithrombotic treatment duration

The optimal duration of antithrombotic therapy in CeAD is an unresolved issue as well. STOP-CAD showed that almost 90% of subsequent ischemic strokes occurred within the first 30 days [50], which was similar to long-term observational data from TREAT-CAD, finding no ischemic events between 3 and 6 months post-CeAD diagnosis [53]. Another study, involving 1,390 patients with a median follow-up of 36 months, observed that the rates of subsequent ischemic cerebral events were comparable between those who received antithrombotic treatment and those who did not (5.0% vs. 4.5%; log-rank test, *P* = 0.53) [54]. These findings suggest an at-risk period, particularly during the first days and weeks following CeAD. TREAT-CAD further reported four hemorrhagic events during the 3–6 month follow-up of 122 participants, pointing towards a potential reversal of the benefit-risk ratio in long-term antithrombotic treatment after CeAD [53]. These findings raise critical questions regarding the optimal duration of therapy, which should be addressed in future CeAD-related trials: How long should antithrombotic treatment be continued in patients with CeAD, and at what point does the risk of hemorrhagic events outweigh the benefit of preventing cerebral ischemic events?

### Individualization of antithrombotic treatment

An additional key question is whether certain subgroups of patients with CeAD are at a higher risk for subsequent ischemic events and may require tailored antithrombotic strategies. Several factors, including occlusion of the dissected artery [55–57], an ischemic cerebral event as the presenting symptom [46, 56–58], and multivessel dissection [56, 59], have been previously associated with subsequent ischemic events as well as worse functional outcomes at three months. A subgroup analysis of the TREAT-CAD trial further substantiated that specific baseline characteristics may increase the risk of recurrent ischemic events and potentially modify the treatment effect of the chosen antithrombotic therapy [60]. Notably, 32 of 33 (97%) primary endpoints—a composite of clinical outcomes (ischemic stroke, severe extracranial or intracranial hemorrhage, death) and MRI-defined new ischemic or hemorrhagic brain lesions—occurred in patients who initially presented with an ischemic cerebral event [60]. Moreover, 16 of 32 (50%) primary endpoints were observed in participants with an occluded dissected artery, despite occlusions being present in only one-third of the cohort [60]. Interestingly, TREAT-CAD and STOP-CAD delivered divergent data on the efficacy of anticoagulation dependent on flow patency in CeAD [50, 60]. In TREAT-CAD, anticoagulation was more beneficial for patients without occlusion [60], whereas in STOP-CAD, it was more beneficial for those with occlusion [50]. However, this discrepancy may be partly explained by differences in outcome measures of the studies: the benefit observed in TREAT-CAD was primarily driven by the inclusion of MRI-based imaging outcomes (i.e., infarcts not accompanied by clinical symptoms, while neuropsychological testing was not performed) [60], whereas STOP-CAD solely reported clinical outcomes (i.e., symptomatic strokes) [50]. While these findings remain exploratory, they highlight the potential for individualized antithrombotic treatment strategies to improve outcomes in patients with high-risk CeAD. This concept has been recognized in the recent American Heart Association (AHA) scientific statement on the treatment and outcomes of CeAD, which proposes an algorithm for optimizing antithrombotic therapy, emphasizing the balance between ischemic risk and the potential for intracranial hemorrhage. [25]

Another potentially relevant factor in disease course and treatment considerations is the presence of dissecting aneurysms, a frequent consequence of CeAD, particularly in patients with multiple dissections [61]. In the CADISS trial, dissecting aneurysms were present in 9.1% of patients at baseline and in 14.5% after three months [62]. While these aneurysms may resolve or decrease in size in about half of patients [25], they can also enlarge or develop de novo over time in both symptomatic and asymptomatic vessels*.* [62] Despite their frequency, dissecting aneurysms generally seem to have a benign prognosis and are reportedly not associated with an increased risk of recurrent stroke [62, 63]. Importantly, their presence does not appear to be influenced by the choice of antithrombotic therapy post-CeAD [62]. As such, they currently should not alter treatment decisions but should remain a consideration in longitudinal imaging follow-up.

## Challenges for a future comprehensive treatment approach

### Vascular risk factors in patients with CeAD – do we have to treat them?

Vascular risk factors have so far received limited attention in the treatment of patients with CeAD. Given the typically young age of these patients, secondary prevention strategies commonly applied to ischemic stroke patients, such as blood pressure lowering, are often underestimated [64], while the usefulness or necessity of statin treatment remains largely unclear. However, several recent observational studies address the role of vascular risk factors in CeAD, offering new insights into their importance.

In terms of atherosclerosis overall, two differing opinions exist in the literature. In a population-based analysis of patients in which CeAD occurred at an older age (65 years or older), an association of CeAD to hypertension, alcohol-, tobacco use, atrial fibrillation, coronary heart disease, and valvular heart disease was found [65]. The authors, therefore, suggested that in those rare cases of CeAD at older age, dissection may arise from acquired vascular injury, primarily due to hypertension and atherosclerosis-related pathology [65]. In contrast, a hospital-based cohort assessing CeAD patients within a typical age range (mean age of 46 years) identified atherosclerotic manifestations in duplex ultrasound in 12% of CeAD patients [66]. This rate was lower than in same-aged healthy controls (healthy females vs. females with CeAD 24% vs. 6%; healthy males vs. males with CeAD 36% vs. 20%) [66, 67]. Moreover, in patients with CeAD and atherosclerotic manifestations, no recurrent CeAD events were observed during a four-year follow-up, compared to 14 recurrent CeAD events in those without atherosclerotic manifestations [66]. These findings suggest that, in contrast to the hypothesis of Kahan et al. [65], who primarily included older CeAD patients, evidence of atherosclerotic manifestations may have a protective effect against recurrent CeAD. Further research into atherosclerosis-related biomarkers, such as lipoprotein(a), and vascular risk factors affecting endothelial dysfunction and thrombosis, including trimethylamine-N-oxide (TMAO), could provide valuable insights into the pathophysiology of CeAD, which have hitherto not been assessed.

Concerning vascular risk factors, Le Grand et al. applied Mendelian randomization analyses to explore the relationship between factors, such as blood pressure, lipid levels, type 2 diabetes, waist-to-hip ratio, smoking, and body mass index, and the incidence of ischemic stroke patients with CeAD and those of stroke with other causes [64]. Their analysis revealed that genetically determined higher systolic blood pressure (OR 1.51, 95% CI 1.32–1.72) and diastolic blood pressure (OR 2.40, 95% CI 1.92–3.00), particularly the latter, significantly increased the risk of CeAD (*p*-value < 0.0001) [64]. These findings strongly support a probable causal relationship between elevated blood pressure and the development of CeAD [64]. Furthermore, their analysis of genetic proxies of drug effects indicated that beta-blockers, should be preferred over calcium channel blockers for managing blood pressure in patients with CeAD. [64]

These studies highlight that, although the role of atherosclerosis in CeAD remains unclear, management of vascular risk factors in CeAD patients is essential. It additionally warrants future research in this to-date rather neglected field of care in patients with CeAD as it holds promise to improve long-term outcomes and reduce the risk of recurrent events.

### Mental health

Mental health in the post-stroke setting has been established as a substantial factor in post-stroke recovery and rehabilitation for years. All-cause ischemic stroke patients report high levels of post-traumatic stress disorder (PTSD), which often relates to the sudden onset of their condition and post-stroke complications [68]. As CeAD patients tend to have favorable outcomes, with about 80% achieving excellent functional status (mRS ≤ 1), it is surprising that they often experience significant mental health burdens [69–72]. Speck et al. reported that PTSD was found in 44% of CeAD patients, compared to just 3% in the general German population [73]. This high prevalence of PTSD was further associated with impaired quality of life, particularly in terms of mental health-related quality of life [73]. It should be noted that this analysis is based on a small sample size including 62 patients, 47 of whom were retrospectively enrolled. [73]

The post-CeAD mental health burden further extends to socioeconomic consequences. A cohort study reported that only 87.6% of previously full-time working CeAD patients returned to work after a median follow-up time of 6.5 years. [71, 74] Women were less likely to return to work compared to men (79.7% versus 93.8%; *p* = 0.010) [74]. Moreover, Mayer-Suess et al. observed higher divorce rates among women during long-term follow-up compared to their male counterparts [74], whereas Fischer et al. found no difference in marital status before and after CeAD [71]. This emphasizes that the clinical outcome assessments currently applied in clinical trials and large observational studies, being primarily dependent on functional outcome and imaging markers, do not paint the entire picture. Therefore, identifying factors and addressing outcomes that truly hamper a return to normal for CeAD patients would have a profound impact on clinical care, emphasizing the need for further research on the matter. [73]

### Screening for CeAD

Currently, there is no evidence to support routine screening for CeAD in asymptomatic individuals, including those with a positive family history or presumed exposure to mechanical strain (e.g., contact or high-impact sports). The role of mechanical triggers, particularly sports-related strain, in the pathophysiology of CeAD remains unclear [2, 9]. Future studies are needed to better define at-risk populations and to evaluate whether targeted imaging could be justified in selected cases.

## Conclusions and future directions

There are unanswered questions in almost all aspects of CeAD care, be it in hyperacute revascularization measures, secondary prevention, or long-term care. As CeAD is the prime cause for ischemic stroke in young adults, this urgently calls for future research which should put special focus on the following:

1. There is a critical clinical need to determine the optimal antithrombotic treatment to prevent subsequent ischemic events while minimizing the risk of bleeding in CeAD patients. Although observational studies have provided valuable insights, a new RCT is essential to definitively answer this question. Contrary to previous assumptions, such a study is now feasible, particularly with the support of the STOP-CAD network, which unites dedicated researchers worldwide. Additionally, recent advances in research integrating Bayesian statistics into new trials allow for the inclusion of patient populations from previous studies, such as TREAT-CAD and CADISS, making this approach particularly valuable for a rare disease like CeAD and enhancing the feasibility of conducting randomized trials in this context.
2. In addition to antithrombotic therapy, it is important to extend research in CeAD treatment to address the importance of vascular risk factors and, especially important from a patient perspective, mental health issues. Further research is needed to better understand their role in the treatment of CeAD and to improve long-term patient care. In this regard, the inclusion of patient-reported outcomes and patient involvement in future studies is of paramount importance, especially in these young CeAD patients, to move closer to an optimal, comprehensive treatment approach and to achieve better outcomes from the patient's perspective.

## Data Availability

Not applicable.
